# Healthy eating index in women with polycystic ovary syndrome: A case-control study

**Published:** 2017-09

**Authors:** Mahbobeh Sadat Hosseini, Alireza Dizavi, Hosein Rostami, Karim Parastouei, Saeed Esfandiari

**Affiliations:** 1 *Clinical Research Development Unit, Baqiyatallah University of Medical Sciences, Tehran, Iran.*; 2 *Department of Clinical Nutrition and Dietetics, Faculty of Nutrition and Food Technology, National Nutrition and Food Technology, Research Institute Shahid Beheshti University of Medical Sciences, Tehran, Iran.*; 3 *Health Research Center, Baqiyatallah University of Medical Sciences, Tehran, Iran.*; 4 *Department of Nutrition, School of Health, Baqiyatallah University of Medical Sciences, Tehran, Iran.*

**Keywords:** Healthy eating index, Polycystic ovary syndrome, Women, Healthy diet

## Abstract

**Background:**

Despite many effort to identify and control the factors involved in polycystic ovary syndrome (PCOS), there are no available reports indicating the association of healthy eating index (HEI) and PCOS.

**Objective:**

The present study has been conducted to examine the relationship between HEI and PCOS.

**Materials and Methods:**

In this case-control study, the study population comprised 297 women aged 20-40 yr referred to Baqiyatallah Hospital Clinics, Tehran, Iran in two groups: case group (n=99) and control group (n=198). The usual dietary data were collected using a validated 168-item semi-quantitative food frequency questionnaire. Standard anthropometric measurements (height, weight, and waist circumference) were also taken. Unconditional logistic regression was used to analyze the relationship between the PCOS and the HEI.

**Results:**

The mean age and body mass index of case and control groups were 29±5.5 vs. 29.5±6 yr and 26.6±4.8 vs. 26±4.2 kg/m², respectively (p=0.752, p=0.822). Mean HEI scores for the case and control groups were found to be 61 and 65, respectively. In final model and after adjustment for confounders, the prevalence of PCOS in subjects in the highest tertile HEI score was significantly (50%) less than those in the lowest tertile HEI score (OR=0.50; 95% CI: 0.25-0.74, p=0.001).

**Conclusion:**

Our results suggest that HEI score is inversely associated with the risk of PCOS in adult women.

## Introduction

Polycystic ovary syndrome (PCOS) is one of the most common endocrinology disorders among women in reproductive age, which involves about 7.8-18.8% of the world’s women population, depending on the diagnostic criteria (1). This syndrome is characterized by heterogeneous symptoms, including the amenorrhea, irregular menstrual cycles, clinical or laboratory evidence of androgen excess, morphological changes, and the presence of antral follicle in the ovaries (2). PCOS is the most common cause of anovulatory infertility, in about 75% of cases (3). The risk of cardiovascular disease, type II diabetes, gestational diabetes, hypertension, lipid disorders, and endometrial cancer increases in women with PCOS (4-6). Although the exact causes of PCOS are not completely understood, current evidence showed that genetics and environmental factors play a role in PCOs pathogenesis (7). Lifestyle interventions have been recommended as first-line treatment in women with PCOs (8). Most of these interventions focus on changing the dietary pattern, physical activity, and weight loss (9, 10). 

A combination of food components incorporated into the appropriate dietary pattern may have more beneficial effects in prevention or control of the disease. Several approaches have been suggested for evaluating the dietary patterns such as healthy eating index (HEI). The HEI was designed to examine the overall quality of diets, its adaptation and compatibility and coordination with dietary guidelines and food pyramid in 1995 (11). This index was designed to evaluate diet quality in different societies with different dietary patterns.

The results of previous studies on the relationship between dietary patterns with PCOS are limited. A survey investigated the Mediterranean dietary pattern among infertile women similar to PCOS and its relationship with the degree of success in achieving natural pregnancy or assisted reproductive technology and observed that compliance with this dietary pattern was associated with increased pregnancy success (12). However, there is no information about the association of HEI-2010 score and PCOS among Iranian female. 

The aim of this study was to determine the relationship between HEI-2010 score and PCOS among Iranian women.

## Materials and methods

**Participants**

This study has been conducted as a hospital-based case-control study of 99 PCOS women and 198 age- and body mass index (BMI)-matched controls. Women in the age group of 19-40 yr reffered to Baqiyatallah Hospital between August 2015 and July 2016 were included in this study. Participants were matched with the control group by age (±5 yr) and BMI (±5 kg/m^2^) (Table I). (We considered, for each 35-year-old woman with bmi=25, three women aged 30-40 yr and bmi 20-30. also should be noted that, were cosidered a large number of the control group (80%) in range of ±2.5 yr and ±2.5 kg/m^2^, and only selected 20% control group in range of ±5 yr and kg/m^2^, respectively). The control group selected from healthy women without any obvious illness. The PCOS was diagnosed on the basis of androgen excess society criteria (1). These criteria recommended that PCOS be defined by three features: 1) androgen excess (biochemical and/or clinical hyperandrogenism), 2) ovarian dysfunction (polycystic ovary morphology and/or oligo-anovulation), and 3) exclusion of other androgen excess or ovulatory disorders. Exclusion criteria were as follows: androgen-secreting tumors, congenital adrenal hyperplasia, Cushing syndrome, thyroid dysfunction, severe insulin resistance syndrome, diabetes, hyperprolactinemia, hypertension, cardiovascular disease, and the use of androgenic or anabolic drugs.

**Assessment of dietary intake**

A validated 168-item food frequency questionnaire was used to assess typical food intake at first examination over the previous year (2). Nutritional information was collected by experienced and trained nutritionist through interviews. Participants reported their intake frequency for each food item during the past year on a daily, weekly, or monthly basis. Portion sizes of eaten food items were then converted to daily grams. Because of incompleteness of Iranian food composition table, energy and other nutrient contents were calculated using the USDA food composition tables (FCT) (14). However, Iranian FCT was used to calculate the nutrients in some foods like Kashk (15). Finally, dietary energy, macronutrients, micronutrients, and food groups were determined.

**Healthy eating index**

HEI comprises 12 components in two main categories; adequacy and moderation. Adequacy includes nine components namely whole grains, total fruits, whole fruits, greens and beans, total vegetables, dairy, total protein foods, seafood, and plant proteins and fatty acid (Polyunsaturated fatty acids [PUFAs]+Monounsaturated fatty acids)/ (Saturated fatty acids). Moderation has three components; as refined grains, sodium, and empty calories. The HEI scoring standard is energy-adjusted (per 1000 kcal) for whole fruits, total fruits, all vegetables, beans, peas, dairy products, meat, seafood, plant proteins, whole grains, refined grains, and sodium. Empty calories; including solid fats and sugar added to food, are calculated as a percentage of total caloric intake. The maximum point value is between 5 and 10 for every component of adequacy category and is between 10 and 20 for those of moderation category. In the adequacy category, higher consumption results in a higher score. If no food from one component is eaten, the component is given a value of zero; if the recommended quantity or more is consumed, the maximum point value is awarded. In moderation category, the maximum point value is attained if the recommended quantity or less is consumed. The original scoring system has 100 points (optimal diet) and gives equal weight to all 10 components (0-10 each) while taking minimal account of age and sex differences. Point values are calculated proportionally for consumed quantities between the maximum and minimum levels of consumption (Table II) (4).

**Anthropometric measurements**

Body weight was measured with a minimal amount of clothing and no shoes on a digital scale (model Seca 803, Germany) to with accuracy of 0.1 kg. Their height was measured using a non-stretchable stadiometer (model Seca 206, Germany) to the nearest 0.1 cm. BMI (kg/m^2^) was calculated as weight (kg) divided by the square of height (m). Waist circumference was measured in the narrowest part between the iliac bones and the lowest rib bones.

**Assessment of other variables**

Baseline information; including age, sex, and medical history were collected using general questionnaires. Physical activity was measured during the interviews with a locally validated version of international physical activity questionnaires. Physical activity was expressed as metabolic equivalents hour/day (METs-h/day) (5, 6).

**Clinical assessments**

Oligomenorrhea/amenorrhea was characterized as fewer than eight menstrual cycles in the previous a year or a menstrual interval of >35 days. Amenorrhea was defined as the absence of menstruation for >3 month. Hyperandrogenism was defined either clinically by hirsutism (modified Ferriman-Gallwey score of ≥8), severe acne or total testosterone level of >80 ng/dL. Polycystic ovarian morphology was identified by pelvic or abdominal ultrasonography and defined as the presence of ≥12 follicles in each ovary. Total testosterone, prolactin, and thyroid-stimulating hormone were measured on day 2-4 of the menstrual cycle. Transvaginal or abdominal ultrasonographic evaluation was performed by experienced sonographers (Table I) (1, 7).

**Ethical consideration**

Written informed consent was obtained from all participants and the study protocol was reviewed and approved by the Human Ethics Committee of Baqiyatallah University (IR.BMSU.REC.1395.247).

**Statistical analysis**

Data were presented as means±SD for continuous variables and as frequencies and percentages for categorical variables. The normality of distribution was calculated using the Kolmogorov-Smirnov test and the histogram curve. The Chi-square test was used to evaluate differences between qualitative variables. Independent t-test or Mann-Whitney U was used to evaluate the differences between continuous variables in normal and abnormal distribution respectively. The HEI score was categorized by tertile. Unconditional logistic regression was used to analyze the association between PCOS and HEI. In all regression analyses, the first tertile was considered as the reference group. To reduce the possibility of spurious associations between the variables, the authors controlled association in two models; Model 1 adjusted for matched factors (age, BMI) and Model 2 made additional adjustments for waist circumference, physical activity, family history of diabetes, education, and energy intake. p≤0.05 was considered as statistically signiﬁcant. Statistical analysis was done using the Statistical Package for the Social Sciences, version 16.0 (SPSS Institute, Chicago, Illinois) (Table III).

## Results

Table I shows the general characteristics of cases (n=99) and controls (n=198). The participation rate was 78% in the case group and 95% in the control group.

Comparing the mean intake between the two groups reveals that the case group has a higher intake of fat (p˂0.001) and carbohydrates (p˂0.001) and lower intake of protein (p=0.05), fiber (p=0.01), and PUFAs (p˂0.001) compared to control group. The mean energy intake in case group was significantly higher than the control group (2600 vs. 2350 kcal/ day) (p=0.01). There was no significant difference between groups in terms of waist circumference, physical activity, or age at menarche (Table I). The mean HEI score and its components in the case and control groups are presented in table II. The mean HEI score in the case group (61) was significantly lower than that in the control group (65) (p=0.001). No significant difference was observed in the scores of beans and peas, all vegetables, empty calories, sodium, total protein, and (PUFAs+Monounsaturated Fatty Acids)/Saturated Fatty Acids) between groups (p>0.05). 

**Figure 1:**
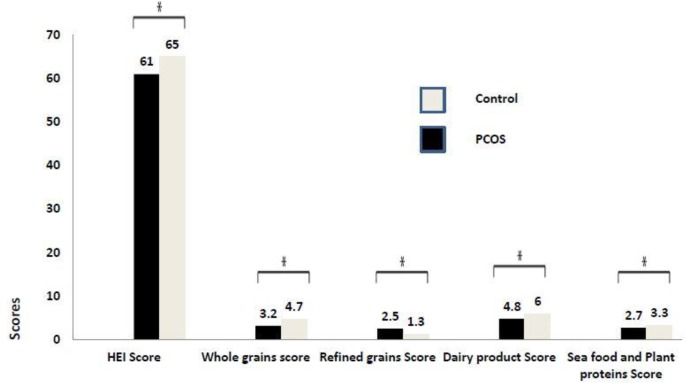
Comparison HEI Score and some of its components between the two groups.

HEI scores for dairy products (p˂0.001), whole grains (p˂0.001), plant proteins and sea foods (p˂0.001) were lower, while refined grains (p˂0.001) was higher in cases than in controls (Figure 1). We observed an inverse association between the risk of PCOS and the HEI score (Table III). In Model 1 adjusted for age and BMI, the odds ratio (OR) of the third tertile compared with the first tertile was 0.49 (OR=0.49, CI 95%, 0.26–0.91, p for trend=0.02). 

**Figure 2:**
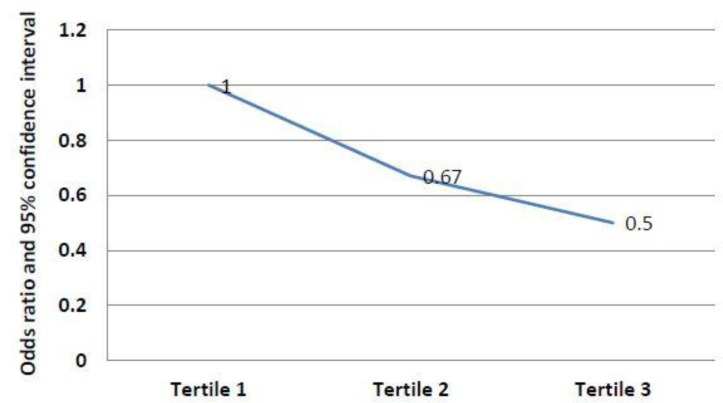
The association of HEI Score with the risk of Polycystic Ovary Syndrome

In Model 2 and after adjustment for all confounding factors such as age, BMI, physical activity, education level, family history of diabetes, and total caloric intake, the PCOS risk further declined for participants who had the healthiest diet (third tertile) based on HEI score, compared to those who had minimum score (first tertile): (OR=0.50, CI 95%, 0.25-0.74, p for trend=0.001) (Figure 2).

**Table I:** Demographic, anthropometric, and clinical characteristics of two study groups

	**PCOS (n=99)**	**Control (n=198)**	* **p value**
Age (yr)	29.0 ± 5.5	29.2 ± 6.0	0.632 **
BMI (kg/m^2^)	26.6 ± 5.0	26.0 ± 4.0	0.852 **
WC (cm)	84.2 ± 12.5	84.5 ± 12.3	0.920 **
Age at menarche (y)	13.2 ± 1.2	13.2 ± 1.4	0.450 **
Educational level, n (%)			0.210 *
Primary school	5 (5.8)	19 (9.5)	
High school	87 (45.5)	40 (40.4)	
Higher education	85 (45.0)	54 (56.5)	
Physical activity (MET-h/day)	59 (42.0)	56 ( 38.0 )	0.820 **
Energy intake (kcal/day)	2600 (892)	2350 (746)	0.010 **
Familial diabetes, n (%)	36 (36.3)	21 (10.6)	0.001 *
Familial hypertension, n (%)	12 (12.3)	22 (11.5)	0.550 *
Familial hyperlipidemia, n (%)	15 (15.2)	28 (14.6)	0.672 *
Vitamin supplement use, n (%)	47 (47.2)	98 (49.9)	0.745 *
Hirsutism, n (%)	52 (52.5)	8 (4.2)	0.001 *
Irregular menstrual cycle, n (%)	60 (60.5)	11 (5.5)	0.001 *
Acne, n (%)	30 (30.6)	10 (5.1)	0.001 *
Amenorrhea, n (%)	10 (10.6)	0 (0.0)	0.001 *
Total testosterone, ng/dl	90 ± 15	-	-
Serum Prolactin, ng/ml	18.1 ± 7.9	-	-
Serum TSH, u/ml	2 ± 0.8	-	-

Data are presented as mean (standard deviation) for continuous variables and number (percent) for categorically distributed variables.

PCOS: polycystic ovary syndrome

BMI: body mass index

WC: waist circumference

MET: metabolic equivalent

* Chi-squared test

** Student’s t test

**Table II:** A comparison between the case and control groups based on scores of the Healthy Eating Index (HEI) and its components

	**Maximum score**	**PCOS (n=99)**	**Control (n=198)**	*p value
HEI scores	100	61.0 (6.5)	65.0 (9.5)	0.001
HEI components				
Whole grains	10	3.2 (3.8)	4.7 (2.9)	0.001
Refined grains	10	2.5 (3.1)	1.3 (2.5)	0.001
Dairy product	10	4.8 (2.6)	6.0 (2.7)	0.001
Total protein foods	5	3.1 (1.0)	3.4 (1.0)	0.080
Sea food and plant proteins	5	2.7 (1.2)	3.3 (1.3)	0.001
Total fruits	5	4.3 (1.1)	4.2 (1.2)	0.048
Whole fruits	5	4.8 (0.5)	4.7 (0.9)	0.066
Total vegetables	5	4.3 (0.9)	4.3 (1.1)	0.780
Greens and beans	5	4.4 (1.1)	4.3 (1.1)	0.480
PUFA:SFA Ratio	10	6.5 (3.8)	5.8 (3.8)	0.160
Sodium	10	6.8 (3.1)	6.8 (3.1)	0.700
SOFAS	20	14.0 (0.5)	15.0 (0.9)	0.640

Data are presented as mean (standard deviation)

PUFA: polyunsaturated fatty acids

SFA: saturated fatty acids

HEI: healthy eating index

SOFAS: calories from solid fat and added sugar

**Table III:** The association of HEI score and its components with the risk of polycystic ovary syndrome

		**Healthy eating index score**			**p-value**
		**T1**	**T2**	**T3**	
	HEI-score	≤ 64	65-72	72≤	
	Crude	1.00	0.94 ( 0.53-1.68 )	0.70 (0.35-0.90)	0.020
	Model 1	1.00	0.93 ( 0.52-1.66 )	0.49 (0.26-0.91)	0.020
	Model 2	1.00	0.67 ( 0.34-1.32)	0.50 ( 0.25-0.74)	0.001
Whole grains					
	Crude	1.00	0.67 ( 0.37-1.50 )	0.38 (0.21-0.60)	0.001
	Model 1	1.00	0.67 ( 0.37-1.27 )	0.37 (0.21-0.70)	0.001
	Model 2	1.00	0.64 ( 0.34-1.23)	0.36 ( 0.19-0.68)	0.002
Refine grains					
	Crude	1.00	1.94 ( 0.71-3.70 )	1.80 (0.80-4.02)	0.650
	Model 1	1.00	1.90 ( 0.69-3.71 )	1.77 (0.82-4.30)	0.600
	Model 2	1.00	1.48 ( 0.60-3.55 )	1.70 (0.72-3.82)	0.680
Dairy					
	Crude	1.00	0.41 ( 0.21-0.74 )	0.29 (0.15-0.54)	0.001
	Model 1	1.00	0.42 ( 0.23-0.75 )	0.28 (0.15-0.54)	0.001
	Model 2	1.00	0.48 ( 0.24-0.89)	0.31 ( 0.16-0.60)	0.001
Total protein foods					
	Crude	1.00	0.61 ( 0.33-1.10 )	0.77 (0.38-1.60)	0.228
	Model 1	1.00	0.63 ( 0.34-1.15 )	0.77 (0.37-1.60)	0.410
	Model 2	1.00	0.70 ( 0.37-1.38)	0.84 ( 0.40-1.78)	0.440
Sea foods					
	Crude	1.00	0.67 ( 0.40-1.14 )	0.24 (0.11-0.51)	0.001
	Model 1	1.00	0.67 ( 0.40-1.25 )	0.24 (0.11-0.53)	0.002
	Model 2	1.00	0.74 ( 0.42-1.30)	0.35 ( 0.18-0.60 )	0.002
Total fruits					
	Crude	1.00	0.71 ( 0.50-1.50 )	0.60 (0.27-1.26)	0.230
	Model 1	1.00	0.70 ( 0.50-1.50 )	0.60 (0.28-1.25)	0.455
	Model 2	1.00	0.60 ( 0.37-1.30)	0.50 ( 0.40-1.10)	0.428
Whole fruits					
	Crude	1.00	0.80 ( 0.62-1.54 )	0.55 (0.28-1.36)	0.324
	Model 1	1.00	0.79 ( 0.61-1.54 )	0.55 (0.28-1.35)	0.470
	Model 2	1.00	0.62 ( 0.30-1.22)	0.45 ( 0.35-1.11)	0.410
Total vegetables					
	Crude	1.00	1.40 ( 0.80-2.26 )	0.77 (0.52-1.70)	0.798
	Model 1	1.00	1.39 ( 0.76-2.20 )	0.76 (0.51-1.69)	0.792
	Model 2	1.00	1.22 ( 0.70-2.15 )	0.71 (0.49-1.60)	0.822
Greens and beans					
	Crude	1.00	1.23 ( 0.74-2.06 )	0.75 (0.50-1.83)	0.821
	Model 1	1.00	1.24 ( 0.72-2.07 )	0.74 (0.50-1.82)	0.798
	Model 2	1.00	1.17 ( 0.70-2.00 )	0.60 (0.45-1.70)	0.806
Sodium					
	Crude	1.00	1.30 ( 0.71-2.40 )	1.25 (0.71-2.20)	0.421
	Model 1	1.00	1.26 ( 0.70-2.40 )	1.18 (0.66-2.11)	0.721
	Model 2	1.00	1.23 ( 0.66-2.31 )	1.11 (0.51-2.81)	0.711
SOFAS					
	Crude	1.00	1.65 ( 0.69-2.60 )	1.81 (0.75-2.85)	0.551
	Model 1	1.00	1.64 ( 0.70-2.40 )	1.82 (0.72-2.84)	0.691
	Model 2	1.00	1.41 ( 0.62-2.29 )	1.22 (0.55-2.79)	0.731

Data are odds ratio and 95% confidence interval

Logistic regression models were used:

Model 1: Adjusted for age (yr) and BMI (Kg/m^2^).

Model 2: Additional adjustment for physical activity (Met-h/day), energy intake (Kcal/d), waist circumference (cm),

the family history of diabetes (yes/no) and education

HEI: healthy eating index

BMI: body mass index

SOFAS: Calories from solid fat and added sugar

T: tertile, T1: poor diet quality

T2: fair diet quality, T3: good

*p˂0.001

## Discussion

In the present matched case-control study, it was observed for the first time that HEI scores had an inverse relationship with the risk of PCOS. We observed that individuals in the highest compared to the lowest HEI scores had 50% lower risk of prevalent PCOS. In case group, 39.5% of women scored “poor”, 37.3% scored “fair”, and only 23.2% scored “good”. Of the adequacy components, approximately, less than 45% of individuals fulfilled the requirement for total vegetables, total fruit, dairy, whole grains, and fatty acids, while greater than 55% fulfilled the requirements for whole foods greens and beans, fruit, total protein, seafood and plant proteins. Few women, on average, fulfilled the recommended restriction levels of the moderation components, sodium (19.9%), empty calories (17.9%) and refined grains (25%) in this population (Table III). 

In the current study, patients with PCOS compared with matched control women had a dietary pattern that was marked by the lower HEI-2010 score. Limited studies are available investigating the association of the HEI-2010 with the prevalence of PCOS. Similar to ours, Rodrigues *et al* which conducted a cross-sectional study on 100 women with PCOS observed that these patients had lower quality diet (including, high intake of sodium, soft drink, refined grains, and low intake of vegetables, fruits, dairy and seafoods) (20). Also, in another study on one hundred sixty-one women with ovulatory disorder who were waiting for assisted reproductive techniques, it was observed that better compliance with the Mediterranean dietary pattern (characterized by high intake of vegetables, vegetable oils, legumes, and fish and low intake of snacks) was associated with increased pregnancy success (12). However, data of 414 PCOS and 7155 non-PCOS subjects from Australian Longitudinal Study on Women's Health population showed that higher adherence to the Mediterranean-style dietary pattern characterized by fish, monounsaturated fats from olive oil, fruits, vegetables, whole grains, legumes, and nuts increase the risk of PCOS (21). This controversy might be because of the quality of dietary intake improved after a diagnosis of PCOS. 

To the best of our knowledge, the current study is the first study that indicated more intakes of whole grains, plant proteins, and seafoods, and fewer intakes of refined grains were associated with lower risk of PCOS. This association remained significant after adjustment for several potential confounders. Also, in our study, women with PCOS consumed considerably fewer dairy products compared to healthy women, and higher intake of dairy was also associated with 69% lower risk of PCOS. The findings of our study about dairy intake are in agreement with the results of previous studies. It has been shown that intake of more than three glasses of milk per day had a protective effect on female fertility (22). In addition, more than two servings of low-fat dairy per day decreased the risk of developing infertility associated with reduced ovulation (23). 

Some mechanisms have been suggested to explain how HEI can be associated with lower risk of PCOS. A possible mechanism is related to the effect of dairy intake on Insulin growth factor-1 (IGF-1) level. Studies suggest that IGF-1 may have a role in ovarian cells disease pathogenesis through the induction of functional changes in the theca cells and higher consumption of dairy products can enhance the IGF-1 level (24, 25). Plant and animal proteins have a differential effect on the IGF-1 level and can play a role in the development of PCOS (24, 26). A positive relationship has been observed between animal proteins and IGF-1 level in women; however, plant protein intake has not been associated with hormone levels (27). Also, it has been documented that insulin resistance decreased in consumers of dairy products, improving ovarian function (28).

In our study, higher intake of whole grains was associated with 64% lower risk of PCOS. The importance of carbohydrate intake due to its effect on the hormonal and metabolic system has been suggested by previous studies. It was indicated that there is a positive relationship between high glycemic index foods and infertility (29), and also there is a beneficial effect of higher intake of whole grains in insulin sensitivity and regulating blood glucose (30).

Several strengths of the current study should be noted. First, the control group was matched based on age and BMI, which partly led to the homogenization of the two groups in terms of physiology and metabolism. Second, the newly diagnosed cases were used to reduce recall bias, since dietary intakes vary with the time of diagnosis due to some diet recommendations as a treatment strategy. Third, the controls were selected among those referred to the same clinic, which makes both groups close in terms of economic and social characteristics.

**Limitation**

The present study had some limitations that should be considered while interpreting the findings. Although a validated FFQ was used for assessment of dietary intakes, however, measurement error and recall bias was unavoidable errors. Also in the current study, we did not have non-hormonal measurements in the control groups. Furthermore, despite controlling for the effects of various confounding variables in the present study, residual confounding due to unknown or unmeasured confounders cannot be excluded. 

## Conclusion

In conclusion, the findings of the current study indicate that high compliance with the HEI-2010 score might be associated with lower risk of PCOS in women. Furthermore, it seems that women with PCOS had lower diet quality compared with matched groups. These findings could be significant, given the increased attention to nutritional therapy in these patients. However, due to low ability of case-control studies in assessing the causality effect, further prospective studies are needed to confirm or refute results of the present study.
